# Impact of COVID-19 on blood donation and transfusion services at Lusaka provincial blood transfusion centre, Zambia

**DOI:** 10.11604/pamj.supp.2020.35.2.23975

**Published:** 2020-06-10

**Authors:** Maisa Kasanga, Steward Mudenda, Theodore Gondwe, Misheck Chileshe, Benjamin Solochi, Jian Wu

**Affiliations:** 1 Zhengzhou University, College of Public Health, 100 Kexue Avenue, Zhengzhou, Henan 450001 China; 2 University Teaching Hospitals, P/Bag RW1X, Lusaka, Zambia; 3 The University of Zambia, School of Health Sciences, Department of Pharmacy, P.O Box 50110, Lusaka, Zambia; 4 The University of Zambia, School of Veterinary Medicine, Department of Disease Control, P.O Box 32379, Lusaka, Zambia; 5 Mary Begg Health Services, 56 Chintu Avenue, Northrise, P.O Box 72221, Ndola; 6 University of Lusaka, Box 36711, Lusaka

**Keywords:** Coronavirus disease 2019, blood donation, transfusion services, Zambia

## To the Editors of the Panafrican Medical Journal

The severe acute respiratory syndrome coronavirus-2 (SARS-CoV-2), the cause of coronavirus disease (COVID-19), was first detected in Wuhan, Hubei Province in China [1]. On 31st December 2019, the World Health Organization (WHO) offices in China were notified of pneumonia of unknown cause [1,2]. Because of the way the disease was spreading, WHO described the outbreak as a Public Health Emergency of International concern on 30th January 2020. On 11th February 2020, WHO officially named it as Coronavirus Disease 2019 (COVID-19) and declared it a pandemic on 11th March 2020. By this time, there were more than 5.4 million COVID-19 cases in 185 countries with 345,000 deaths [1,3]. On 18th March, Zambia recorded its first 2 COVID-19 cases [4]. Since then, COVID-19 cases have continued increasing in Zambia [5].

**Figure 1:**
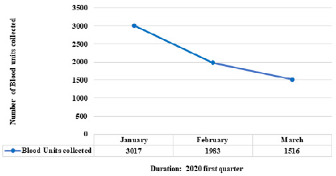
Trends of blood donation in the first quarter

The University Teaching Hospitals (UTH) houses the Lusaka Provincial Blood Transfusion Centre (LBTC). The LBTC collects blood in two main ways: through mobile outreach (in schools, colleges, and universities) and at the fixed site (walk-in donors and family members of in-patients). Before the COVID-19, the centre collected approximately 90% of blood from mobile outreach and 10% from the fixed site. First-quarter of 2020 [mean = 2172, SD = 768; Range: 264-4080] compared to the 2019 [mean = 3446, SD = 703; Range: 1700-5192], even though this decrease is not statistically significant (p = 0.50). The target for the first quarter of 2019 was 11, 250 units of blood, but 10, 338 (91.9%) was collected. In the 2020 first quarter, the target was 18, 750, and only 6, 516 units (34.7%) was collected, way below the target. Interestingly, it occurred around February and March when people knew of COVID-19 as shown in Figure 1. Blood collection during the COVID-19 pandemic has reduced because people believe that by donating blood, they could contract COVID-19. Secondly, when the partial lock-down was declared, all learning institutions were closed causing mobile blood collection to cease. The closure of learning institutions was done to avoid the spread of COVID-19 in communities through social interaction [6]. Health workers were among the donors to reduce the shortfall, but due to an increased number of COVID-19 cases, most of them went to the front line. Walk-in blood donors no longer have easy access to the centre due to strict measures put in place to prevent further spread of COVID-19.

The primary beneficiaries of the blood are expectant mothers, paediatric, renal, and cancer sections. Therefore, if the COVID-19 pandemic continues, the trend of blood donation is likely to continue, and this will negatively impact the blood beneficiaries [7]. Interventions to mitigate blood shortage due to COVID-19 are; Sensitization of communities on the transmission and spread of COVID-19, and the importance of donating blood. Assurance of security safety measures for giving blood donation without risking one’s health. Cooperating partners such as banks and other organizations should encourage their workers to donate blood. Some farming blocks away from epicentres of COVID-19 must be identified for mobile outreach. These mobile outreach initiatives must take into account precautions, such as ascertaining the safety of donors in terms of their exposure and travel history to any COVID-19 hotspots. Measures have been put in place to work smoothly with the security to allow the walk-in donors visit the centre.

The challenge posed by COVID-19 on blood donation is real. This calls for measures that would ensure prudent use of donated blood and thereby maintain transfusion services. Some of the following recommendations could be applied during the COVID-19 era: Use of pharmacological agents such as desmopressin in the treatment of mild haemophilia instead of transfusing blood [8]. Similarly, vasoconstrictor agent aprotinin can be used to reduce the loss of blood from the operative site during surgery. Fluid replacement and use of volume expanders such as crystalloids or colloids have should be encouraged during this COVID-19 pandemic, especially in Zambia. Hematinics for patients with anaemia should be viewed as a better option instead of blood transfusion, so that blood for transfusion should be reserved for patients in real need [9]. A well-organized communication with donors should be established. For example, mobile phone messages should be sent to people in the community in association with mobile service providers so that those who might be willing to donate can easily do so. Blood donors should be given appointment letters encouraging them to visit blood centres and donate blood during the lock-down period [10]. This will help facilitate adequate blood donation.

In conclusion, the impact of COVID-19 on blood collection and transfusion services can surely be felt at LBTC. We hope that despite the pandemic, more and more people will see the need to donate blood to meet the many challenges of healthcare provision in Zambia. A multisectoral approach is recommended to curb the pandemic and the problems it poses.

## Competing interests

The authors declare no competing interests.
